# Advancing Pediatric Surgery: The Use of HoloLens 2 for 3D Anatomical Reconstructions in Preoperative Planning

**DOI:** 10.3390/children12010032

**Published:** 2024-12-28

**Authors:** Marco Di Mitri, Annalisa Di Carmine, Simone D’Antonio, Benedetta Maria Capobianco, Cristian Bisanti, Edoardo Collautti, Sara Maria Cravano, Francesca Ruspi, Michele Libri, Tommaso Gargano, Mario Lima

**Affiliations:** Pediatric Surgery Department, IRCCS Azienda Ospedaliero, Universitaria di Bologna, Via Massarenti 11, 40138 Bologna, Italy; annalisa.dicarmine@studio.unibo.it (A.D.C.); simone.dantonio@aosp.bo.it (S.D.); bendetta.capobianco@studio.unibo.it (B.M.C.); cristian.bisanti@studio.unibo.it (C.B.); edoardo.collautti@studio.unibo.it (E.C.); sara-cravano@libero.it (S.M.C.); francesca.ruspi@studio.unibo.it (F.R.); michele.libri@aosp.bo.it (M.L.); tommaso.gargano2@unibo.it (T.G.); mario.lima@unibo.it (M.L.)

**Keywords:** HoloLens 2, three-dimensional reconstruction, Verima, pediatric surgery, innovations, mixed reality, augmented reality, preoperative planning

## Abstract

Background: In pediatric surgery, a comprehensive knowledge of the child’s anatomy is crucial to optimize surgical outcomes and minimize complications. Recent advancements in medical imaging and technology have introduced innovative tools that enhance surgical planning and decision-making. Methods: This study explores the integration of mixed reality technology, specifically the HoloLens 2 headset, for visualization and interaction with three-dimensional (3D) anatomical reconstructions obtained from computed tomography (CT) scans. Our prospective observational study, conducted at IRCCS (Scientific Hospitalization and Care Institute) Sant’Orsola-Malpighi University Hospital in Bologna, engaged ten pediatric surgeons, who assessed three types of anatomical malformations (splenic cysts, pulmonary cystic adenomatoid malformations, and pyelo-ureteral junction stenosis) and planned surgeries using both traditional 2D CT scans and 3D visualizations via HoloLens 2, followed by completing a questionnaire to evaluate the utility of each of these imaging techniques in surgical planning. Results: The statistical analysis revealed that the 3D visualizations significantly outperformed the 2D CT scans in clarity and utility (*p* < 0.05). The results indicated significant improvements in anatomy understanding and surgical precision. The immersive experience provided by HoloLens 2 enabled surgeons to better identify critical landmarks, understand spatial relationships, and prevent surgical challenges. Furthermore, this technology facilitated collaborative decision-making and streamlined surgical workflows. Conclusions: Despite some challenges in ease of use, HoloLens 2 showed promising results in reducing the learning curve for complex procedures. This study underscores the transformative potential of mixed reality technology in pediatric surgery, advocating for further research and development to integrate these advancements into routine clinical practice.

## 1. Introduction

In the field of pediatric surgery, the ability to comprehensively understand and manage the child’s anatomy is essential for achieving optimal surgical outcomes while minimizing complications. In recent years, there have been significant advancements in medical imaging and technology, offering surgeons innovative tools to improve their preoperative planning and intraoperative decision-making process [1,2,3]. Among these transformative innovations, the integration of mixed reality technology, in particular the HoloLens 2 headset, has emerged as a groundbreaking solution for visualizing and interacting with three-dimensional (3D) reconstructions derived from computed tomography (CT) scans [4]. The utilization of 3D scan reconstructions through HoloLens 2 represents a paradigm shift in surgical planning. By seamlessly overlaying virtual anatomical structures onto the real-world surgical environment, surgeons are provided with an unprecedented level of spatial awareness and depth perception. This immersive visualization capability goes beyond the traditional two-dimensional representations, allowing surgeons to gain a holistic understanding of anatomical relationships, vascularization patterns, and the dimensions of pathological findings. One of the main advantages of using mixed reality technology in pediatric surgical planning is its ability to enhance comprehension of the anatomical structures. By immersing themselves in a virtual representation of the patient’s anatomy, surgeons can navigate through complex structures with unmatched precision and clarity [5,6]. This technology enables them to identify critical anatomical landmarks, assess the spatial relationships between structures, and anticipate potential challenges or complications that may arise during the surgical procedure. Furthermore, the ability to interact with the 3D reconstructions in real time facilitates collaborative decision-making among members of the surgical team, thereby promoting a multidisciplinary approach to patient care. The integration of HoloLens 2 into the surgical workflow has been shown to improve surgical precision and efficiency as well as enhance anatomical comprehension. With the ability to visualize the intended surgical approach and simulate intraoperative maneuvers in a virtual environment, surgeons can more effectively plan their surgical strategy and optimize treatment outcomes. Moreover, the integration of augmented reality overlays directly into the surgical field allows surgeons to maintain focus on the patient while concurrently accessing critical information and guidance, thereby minimizing interruptions and streamlining workflow.

### Aim of This Study

Therefore, the aim of this study is to analyze the advantages of 3D reconstructions visualized using HoloLens 2 mixed reality viewer in preoperative planning, comparing it with the use of 2D CT images, by means of a questionnaire administered to pediatric surgeons.

## 2. Methods

**Figure 1:**
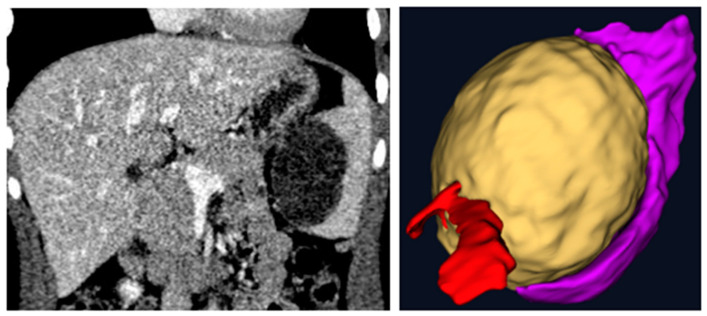
Two-dimensional vs three-dimensional: a splenic cyst (yellow) with emphasis on its relationships with the vascular hilum (red) and on its compression of the spleen (purple).

**Figure 2:**
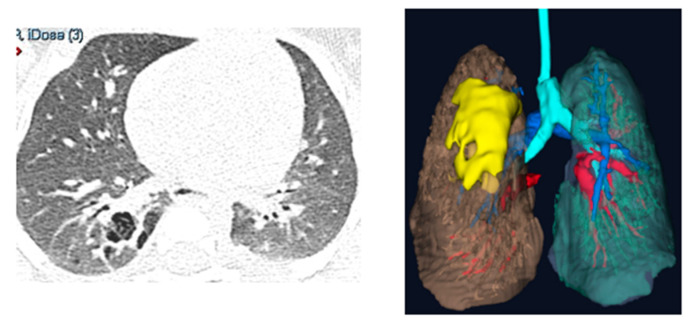
Two-dimensional vs three-dimensional: the reconstruction of a pulmonary cystic adenomatoid malformation (yellow) highlighting relationships with the surrounding lung parenchyma (brown and light blue) and vessels (red and blue).

**Figure 3:**
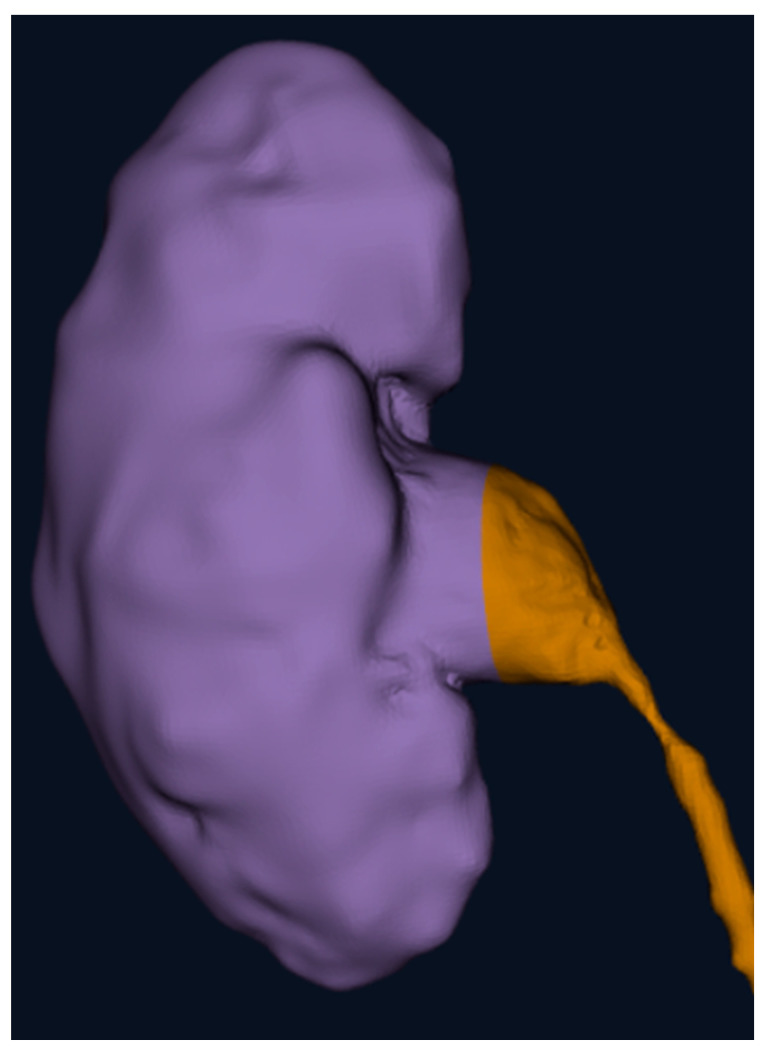
The 3D reconstruction of a kidney (purple) with a stenosis of the pyelo-ureteral junction (brown) focusing on the position of the pelvis and ureter and on the stenosis’ characteristics.

A prospective observational study was conducted in our Department of Pediatric Surgery at IRCCS Sant’Orsola-Malpighi University Hospital in Bologna. Our study was conducted in accordance with the principles outlined in the Declaration of Helsinki. Ethical review and approval were waived for this study because it did not involve experimental procedures or interventions and because of the small number of patients involved. Additionally, informed consent was obtained from all patients for the use of their radiological findings in this research. The data were anonymized to ensure confidentiality and compliance with ethical standards. Ten pediatric surgeons from our team were enrolled. Each surgeon evaluated three types of anatomical abnormalities: a splenic cyst (n = 1) (Figure 1), a pulmonary cystic adenomatoid malformation (n = 1) (Figure 2), and a stenosis of the pyelo-ureteral junction (n = 1) (Figure 3). For each of the three types of pathologies, the surgeons first reviewed traditional two-dimensional (2D) computed tomography (CT) scans and then three-dimensional (3D) reconstructions visualized using the HoloLens 2 mixed reality viewer. After these evaluations, the surgeons completed a questionnaire to compare the utility of the 2D CT scans and the 3D visualizations for surgical planning.

All surgeons involved in this study had at least 5 years of experience in the treatment of these pathologies.

**Figure 4:**
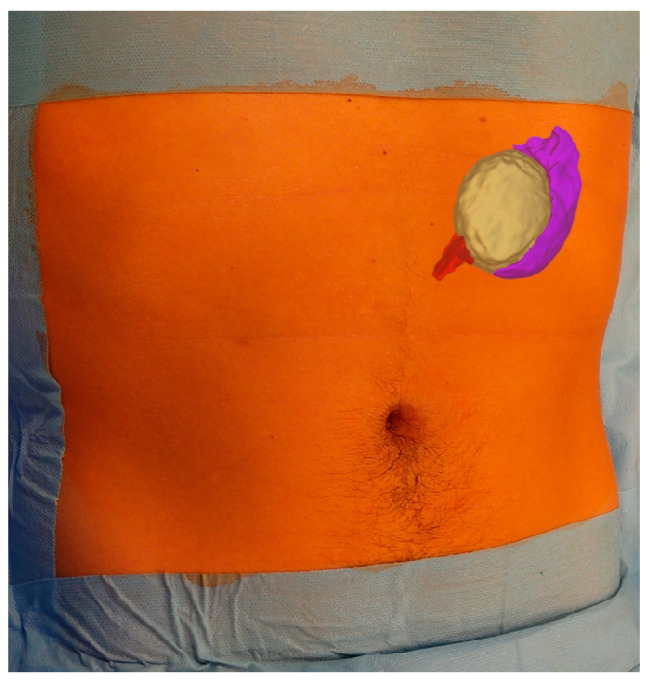
Application of the augmented reality by HoloLens 2 on the patient’s body, enhancing the surgeon’s skill to identify the position of the cyst (yellow), its relationships with the spleen (purple), and the vascular hilum (red) before the incision.

The DICOM file of the CT was uploaded in Verima software (Witapp AR) (https://verima.it) and then processed in order to create the 3D visualizations. We applied HoloLens 2 technology in the operating room, projecting the 3D images, downloaded on the Verima app (available on the Google Play Store), on the patient’s body before the surgery (Figure 4).

Samples:

- *Patient 1*: affected by pulmonary cystic adenomatoid malformations, 8 months old, 10 kg weight, no other comorbidity.
- *Patient 2*: affected by a splenic cyst of 6 cm, 16 years old, 82 kg, no other comorbidity.
- *Patient 3*: affected by a stenosis of the pyelo-ureteral junction, 7 years old, 31 kg, no other comorbidity.

### 2.1. Verima Software

The Verima software is a comprehensive tool used for creating detailed 3D reconstructions from CT scans, greatly enhancing the accuracy and effectiveness of surgical planning [7,8,9]. The process begins with the Verima Tool, which allows medical professionals to segment anatomical structures from DICOM files, facilitating the creation of precise 3D models. These models provide a clear and comprehensive view of the patient’s anatomy, which is particularly advantageous in pediatric surgery, where anatomical variations can be significant. Subsequently, the generated 3D models can be displayed using the Verima Viewer application on Microsoft HoloLens 2. This mixed reality visualization immerses surgeons in a holographic environment where they can interact with the models in real time. The possibility to rotate, zoom, and manipulate the holograms facilitates the understanding of complex anatomical relationships and the planning of intricate surgical procedures with greater precision. This combination of Verima software and HoloLens 2 not only improves preoperative planning but also enhances intraoperative guidance, thereby potentially reducing surgical risks and improving outcomes in pediatric patients.

### 2.2. HoloLens 2

HoloLens 2, developed by Microsoft, is a cutting-edge mixed reality device that seamlessly blends the physical and digital world, providing an immersive augmented reality experience. With its advanced sensors, high-resolution displays, and intuitive hand-tracking capabilities, HoloLens 2 allows users to interact with 3D holograms in their environment as if they were real objects. This technology has significant applications in various fields, including medicine, where it can revolutionize surgical planning and execution. For instance, in pediatric surgical procedures, HoloLens 2 can overlay 3D models of a patient’s anatomy into the surgeon’s field of view, enabling precise preoperative planning and intraoperative guidance. This enhanced viewing helps with understanding complex anatomical structures and spatial relationships, which is particularly useful for pediatric patients whose anatomy may exhibit significant variability. By providing real-time, interactive visualizations, HoloLens 2 helps to improve surgical accuracy, reduce risks, and potentially enhance outcomes in pediatric surgery [4,10,11].

In our study, we used augmented reality with HoloLens 2 to show the 3D reconstruction directly on the patient’s body before surgery. This application is useful because it can lead the surgeon to have a preliminary image of the intraoperative findings. Therefore, the use of augmented reality with HoloLens 2 allows the surgeon to choose the correct position of the trocars and be able to know the anatomical and vascular relationships of the pathological findings to be treated before the surgical procedure.

### 2.3. Questionnaire

**Figure 5:**
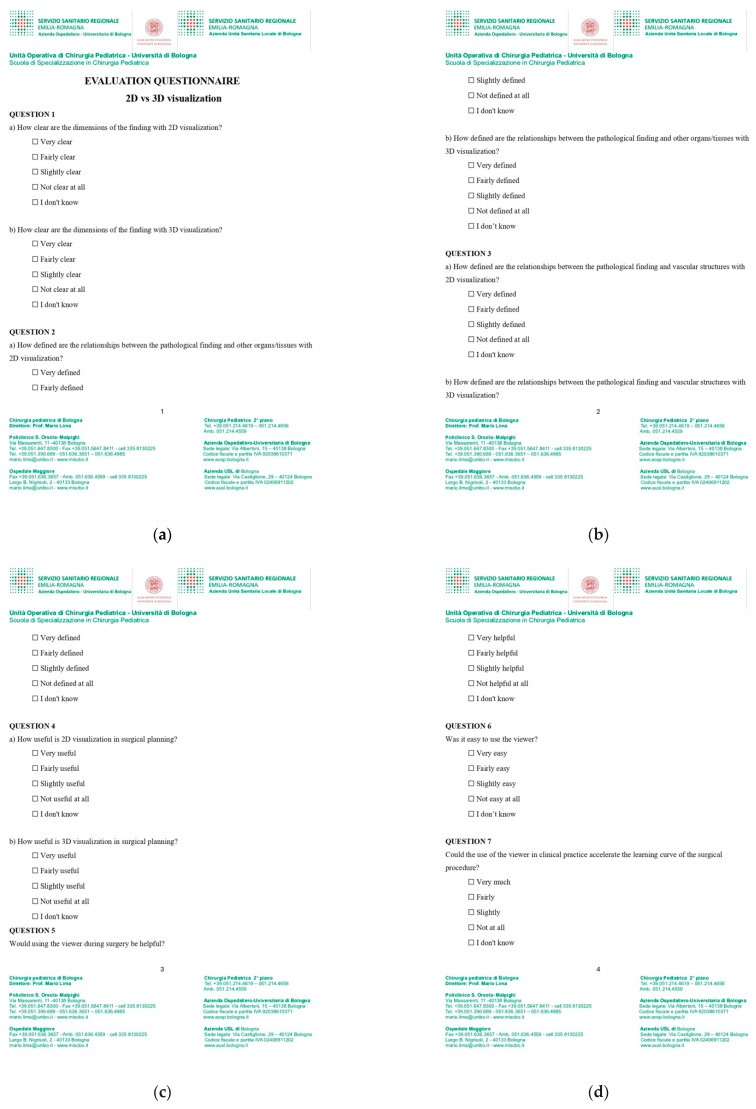
(**a**–**d**) Questionnaire.

The administered questionnaire (Figure 5) was created using the Likert rating scale, which allows the measurement of users’ opinions and perceptions. The ability to evaluate subjective elements comes from the use of a graduated scale that allows users to express their opinions, offering more nuanced answers than a simple yes/no binary. Through the proposed questions, we were able to gather information regarding the efficacy of augmented reality in defining the anatomical features of pathological findings and selecting the most suitable surgical technique for patients, as well as in potentially reducing the surgical times and the learning curve of surgical procedures. Furthermore, the questionnaire investigated the ease of use of the viewer.

### 2.4. Statistical Analysis

The statistical analysis involved comparing paired responses for usability and clinical utility (dependent variables) between 2D CT scans and 3D visualizations obtained via HoloLens 2 (independent variable) using paired *t*-tests. *p*-values were calculated to assess statistical significance, with normality confirmed via the Shapiro–Wilk test prior to the analysis. Additionally, Cohen’s d was used to measure effect sizes, providing a quantifiable assessment of the magnitude of these differences.

The data were analyzed using the following steps:

1. Calculate the mean and standard deviation for the responses to each question for both the 2D and 3D visualizations.
2. Perform the paired *t*-tests for each question, as follows:
  - Question 1: 2D vs. 3D
  - Question 2: 2D vs. 3D
  - Question 3: 2D vs. 3D
  - Question 4: 2D vs. 3D
3. Report the *t*-statistic and *p*-value for each comparison to determine statistical significance.

## 3. Results

Paired *t*-tests indicate that for questions 1 through 4, 3D visualization using HoloLens 2 significantly improves surgeons’ ability to view preoperative CT images and therefore enhances the interpretation of pathology dimensions as well as the relationships with the surrounding organs and vessels, improving preoperative surgical planning (Table 1).

The results revealed a large effect size (d = 1.15) for usability scores, indicating a substantial improvement with 3D visualizations. For surgical time, the effect size was also large (d = 1.4), underscoring the efficiency gains with HoloLens 2 technology. In contrast, the effect size for blood loss was small (d = 0.3), suggesting minimal clinical impact on this parameter. These findings highlight the transformative potential of mixed reality in enhancing surgical precision and planning.

Surgeons highlighted the potential benefits of using HoloLens 2 as an additional tool during surgical decision-making, with an average rating of 4.5 (±0.5). They also acknowledged that this technology could reduce the procedure-specific learning curve, giving it an average rating of 4.3 (±0.6). However, not all surgeons found the device to be easy to use, with this aspect receiving a lower average rating of 2.9 (±0.9) (Table 2).

A subgroup analysis was performed to investigate whether age, gender, or years of experience influenced attitudes toward the use of HoloLens 2. Responses to the Likert-scale questionnaire were stratified by these demographic variables and analyzed using an independent *t*-test for gender and experience groups (junior vs. senior surgeons) and a Pearson correlation test for age (Table 3). The analysis revealed no statistically significant differences in perceptions of utility or ease of use based on these factors (*p* > 0.05 for all comparisons).

## 4. Discussion

The integration of HoloLens 2 mixed reality technology in pediatric surgical planning represents a significant advancement in this field. This study aimed to assess the efficacy of 3D visualization using HoloLens 2 in comparison to traditional 2D CT scans, focusing on enhancing the understanding of anatomical structures and improving surgical decision-making.

### 4.1. Enhanced Definitions of Anatomical Dimensions, Relationships, and Vascular Structures

Our results indicate that the clarity of anatomical dimensions was significantly improved with the 3D visualizations. Surgeons rated the clarity of dimensions higher for the 3D visualizations compared to the 2D CT scans (*p* < 0.01). This enhancement is critical for preoperative planning as it allows surgeons to more accurately appreciate the size and spatial relationships of pathological findings, which is frequently challenging with 2D images.

The ability to define the relationships between pathological findings and the surrounding tissues is crucial in surgical planning. Our study found that 3D visualization significantly improved the definition of these relationships compared to 2D CT scans (*p* < 0.01). This improvement enables surgeons to plan more precise surgical interventions, potentially reducing the risk of intraoperative complications and improving surgical outcomes.

Understanding the relationship between pathological findings and vascular structures is essential for preventing vascular injuries during surgery. The 3D visualizations provided by HoloLens 2 were rated significantly higher than the 2D CT scans (*p* < 0.01) in terms of defining these relationships. The enhanced visualization of vascular structures facilitates meticulous surgical planning and execution, thereby contributing to safer surgical practices.

### 4.2. Utility in Surgical Planning and Intraoperative Application

The perceived utility of 3D visualization in surgical planning was significantly greater compared to 2D CT scans (*p* < 0.01). Surgeons reported that the 3D models provided a more comprehensive understanding of the anatomical scenario, leading to the development of better-informed surgical strategies. This finding underscores the potential of mixed reality technology to enhance the overall quality of surgical planning. For instance, the ability to overlay virtual anatomical structures onto the real-world surgical environment allows surgeons to accurately map out incision sites and trajectories, minimizing tissue trauma and optimizing surgical access [12,13]. Furthermore, the immersive nature of HoloLens 2 facilitates the integration of patient-specific data, such as preoperative imaging and physiological parameters, into the surgical planning process. This personalized approach to surgical decision-making enhances precision and tailors interventions to individual patient needs, ultimately improving surgical outcomes and patient satisfaction [14,15].

When considering the potential use of HoloLens 2 during surgery, surgeons indicated a high level of perceived benefit (mean score: 4.6). The ability to interact with 3D models in real time provides dynamic guidance and can help in making critical decisions during surgical procedures. This real-time interaction is particularly advantageous in complex pediatric cases where anatomical variations are significant.

### 4.3. Ease of Use and Learning Curve

Surgeons rated HoloLens 2 as relatively easy to use (mean score: 4.4), suggesting that the integration of this technology into clinical practice can be smooth with adequate training. Furthermore, the use of HoloLens 2 is perceived to potentially accelerate the learning curve for surgical procedures (mean score: 4.5), which could be particularly advantageous for trainee surgeons.

### 4.4. Collaboration and Multidisciplinary Approach

Expanding on the theme of collaboration, the integration of HoloLens 2 technology fosters synergistic interactions not only within the surgical team but also across disciplines and institutions. Telemedicine platforms enabled by HoloLens 2 facilitate real-time consultations and remote surgical mentoring, overcoming geographical barriers and promoting knowledge exchange among healthcare professionals worldwide. Moreover, the possibility to share three-dimensional reconstructions and surgical plans in a virtual environment enhances interdisciplinary communication and fosters a culture of continuous learning and innovation. By leveraging the collective expertise of diverse stakeholders, we can collectively tackle complex surgical challenges and advance the frontiers of pediatric surgical care [16,17,18].

### 4.5. Contextualization of the Results

The statistical results of this study highlighted that surgeons rated the clarity of dimensional information from 3D visualizations as superior to that from 2D CT scans, a statistically significant difference with practical implications for preoperative planning. This improvement is essential for surgeons to accurately assess the size and spatial relationships of pathological findings, which directly influences surgical precision. This study showed that 3D visualizations significantly outperformed 2D CT scans in delineating the relationship between pathological findings and vascular structures, which is crucial for preventing vascular injuries during surgery. Surgeons perceived 3D visualization as far more useful in surgical planning, as it provided a comprehensive understanding of the anatomical context. This facilitated the creation of more precise surgical strategies, such as accurately mapping incision sites and optimizing surgical trajectories, thereby minimizing tissue damage and improving surgical incision accuracy. Furthermore, the immersive nature of HoloLens 2 also enhanced teamwork, real-time decision-making, and workflow efficiency, making it easier for surgeons to collaborate and share insights. These factors suggest that 3D visualization technologies not only improve surgical planning but also promote better outcomes through improved team dynamics and communication.

Although some usability challenges remain, HoloLens 2 also demonstrated significant promise in reducing the learning curve for complex pediatric procedures. This 3D visualization technology could be functional for improving young surgeons’ skills in planning and performing surgical procedures, accelerating their learning process. Despite the lack of studies in the literature on structured surgical training programs, HoloLens 2 technology seemed to provide encouraging features in educating young surgeons [18].

Although numerous improvements are needed in the application of 3D technology in clinical practice, several studies have been conducted on the use of 3D visualization through HoloLens in various fields of surgery.

H. Brun et al. [4] performed a study with the aim of investigating the utility of mixed reality holograms in heart surgical planning, based on 3D heart models from CT angiograms. They demonstrated, with a questionnaire administered to 36 surgeons, that 3D holograms could be a surgical planning tool for congenital heart disease.

A comprehensive literature search was conducted by Saini et al. [15] across relevant databases to identify studies published utilizing 3D imaging techniques and virtual patients, focusing on the accuracy of dental implant planning and surgical placement. The synthesis of the available evidence highlighted the substantial positive impact of 3D imaging techniques and virtual patients on dental implant planning and surgical placement accuracy, contributing to a more personalized and precise approach that enhances overall treatment outcomes.

Gsaxner et al. [19] conducted a systematic review analyzing 217 publications about different applications of HoloLens technology in medical fields such as surgical planning, medical simulators, image-guided interventions (especially in orthopedic surgery and neurosurgery), and rehabilitation programs of patients. They observed that a large proportion of studies focused on preoperative imaging and planning data. When it comes to data and visualization, the majority of studies displayed preoperatively acquired 3D medical imaging data, primarily from CTs or CTAs, visualized through surface rendering, which is easy to create and modify. Despite increased efforts in the areas of precision, reliability, usability, and workflow, more studies are still necessary to establish this technology in clinical practice. However, the 3D visualization models provided a more comprehensive understanding of the anatomical scenario, leading to the development of better-informed surgical strategies in the preoperative workup.

### 4.6. Limitations of This Study

We are aware of the limits of this study. In the first place, the small number of surgeons involved and cases analyzed. Secondly, the limitation of the applications of HoloLens 2 technology in the preoperative phase. Nevertheless, we believe our data could provide an interesting and encouraging preliminary basis for further investigations.

### 4.7. Challenges and Future Directions

Despite the immense potential of HoloLens 2 technology, several challenges must be addressed to facilitate its widespread adoption and integration into clinical practice. Technical hurdles, including interoperability issues and data security concerns, require robust infrastructure development and regulatory oversight to protect patient privacy and ensure seamless integration with existing healthcare systems. Moreover, disparities in access to technology and training must be mitigated to prevent exacerbating existing healthcare inequalities. Collaborative efforts between policymakers, industry leaders, and healthcare providers are crucial for overcoming these barriers and promoting equitable access to advanced surgical technologies.

Looking ahead, the future of pediatric surgical innovation hinges on harnessing the synergistic potential of emerging technologies, interdisciplinary collaboration, and patient-centered care. As we navigate the complexities of the digital age, it is imperative to uphold ethical principles, prioritize patient safety, and sustain humanistic values in the pursuit of surgical excellence. By embracing a culture of innovation and inclusivity, we can leverage the transformative power of HoloLens 2 technology to redefine the standard of care in pediatric surgery and usher in a new era of precision medicine.

## 5. Conclusions

The adoption of HoloLens 2 for 3D visualization in pediatric surgical planning offers significant advantages over traditional 2D CT scans. The enhanced clarity of anatomical structures, the improved definition of anatomical and vascular relationships, and the perceived utility in both preoperative planning and intraoperative guidance highlight the transformative potential of this technology. By facilitating a more precise and informed approach to surgery, HoloLens 2 holds promise for improving surgical outcomes and advancing the standard of care in pediatric surgery. Further research and clinical trials will be essential in solidifying its role in surgical practice.

## Figures and Tables

**Table 1:** Comparison between the 2D and 3D visualizations.

Question	Comparison	*t*-Statistic	*p*-Value
1	2D vs. 3D	−75.243	<0.01
2	2D vs. 3D	−107.012	<0.01
3	2D vs. 3D	−78.657	<0.01
4	2D vs. 3D	−103.923	<0.01

**Table 2:** Surgeon’s point of view about the role of HoloLens 2 in surgical planning.

Question	Main	DS
5	4.5	±0.5
6	2.9	±0.9
7	4.3	±0.6

**Table 3:** Surgeons’ demographic data.

Surgeon ID	Sex	Age	Experience
1	M	35	Junior
2	F	46	Senior
3	M	37	Junior
4	F	50	Senior
5	M	50	Senior
6	M	38	Junior
7	F	47	Senior
8	M	46	Senior
9	F	52	Senior
10	M	48	Senior

## Data Availability

The data presented in this study are available upon request from the corresponding author due to privacy restrictions.
